# Comparative evaluation of biologically and chemically synthesized zinc oxide nanoparticles for preventing *Candida auris* biofilm

**DOI:** 10.1007/s10534-025-00678-6

**Published:** 2025-03-31

**Authors:** Bahgat Fayed, Hoda S. El-Sayed, Shanshan Luo, Aisha E. Reda

**Affiliations:** 1 Chemistry of Natural and Microbial Product, National Research Centre, Dokki, Cairo, 12622 Egypt; 2 Dairy Department, National Research Centre, Dokki, Cairo, 12622 Egypt; 3 Institute of Hematology, Union Hospital, Tongji Medical College, Huazhong University of Science and Technology, 1277 Jiefang Avenue, Wuhan, 430022 China; 4 Refractories, Ceramics and Building Materials Department, National Research Centre, Dokki, Cairo, 12622 Egypt

**Keywords:** *Candida auris*, Zinc oxide nanoparticles, Biofilm, Antifungal resistance

## Abstract

*Candidozyma auris* (formerly *Candida auris*) is a multidrug-resistant yeast that poses a significant global health threat due to its ability to form biofilms and resist various antifungal treatments. This study evaluates and compares the antifungal efficacy of biologically synthesized zinc oxide nanoparticles (ZnO-NP-B) and chemically synthesized ZnO nanoparticles (ZnO-NP-C1 and ZnO-NP-C2), developed using the dry–wet chemical method and sol–gel method, respectively. ZnO-NP-B was synthesized using *Lactobacillus gasseri*. The nanoparticles were characterized for size, charge, and morphology using Particle Size Analyzer, photon correlation spectroscopy with a 90 Plus Zetasizer, and scanning electron microscopy (SEM), respectively**.** The antifungal activity was assessed through minimum inhibitory concentration (MIC_50_) determination, biofilm inhibition assays by XTT assay, and gene expression analysis. ZnO-NP-C1 exhibited the highest antifungal activity against *C. auris* planktonic cells, with a MIC_50_ value of 61.9 ± 3.3 µg/ml, followed by ZnO-NP-C2 (151 ± 7.83 µg/ml), whereas ZnO-NP-B showed limited efficacy (MIC_50_ = 1 mg/ml). Chemically synthesized ZnO-NPs, particularly ZnO-NP-C2, did not induce overexpression of resistance genes (*CDR1, MDR1, ERG2, ERG11, FKS1, CHS1*), whereas ZnO-NP-B triggered their upregulation, potentially promoting resistance. ZnO-NP-C1 was the most effective in preventing biofilm formation, reducing *C. auris* adhesion by 67.9 ± 2.35% at 150 µg/ml, while ZnO-NP-B exhibited negligible inhibition. Gene expression analysis further confirmed that ZnO-NP-C1 significantly downregulated adhesive genes (*ALS5, IFF4, CSA1*) by up to 0.37 ± 0.006, 0.043 ± 0.002, and 0.06 ± 0.0004, respectively. These findings highlight the potential of ZnO-NP-C1 as a promising antifungal agent for preventing *C. auris* biofilms, emphasizing the critical role of synthesis methods in optimizing nanoparticle properties for antifungal applications.

## Introduction

*Candidozyma auris* (formerly *Candida auris*) (*C. auris*) is multi-drug resistant yeast that become a major global health concern, especially in healthcare environments, because of its rapid spread and high level of resistance to multiple drugs (Schelenz et al. 2016). *C. auris* has caused numerous outbreaks worldwide with elevated mortality rate, particularly in patients with deficient immune systems (Fayed et al. 2023). What sets *C. auris* apart from other fungi is its ability to resist several types of antifungal drugs. This extreme level of resistance makes treatment options for infections caused by these strains exceptionally limited and poses a severe challenge in clinical settings. The emergence of pan drug resistant *C. auris* is provoked by its ability to evolve under antifungal pressure, especially in regions with widespread use of antifungal agents (Ademe et al. 2020). Treatment options are largely experimental or based on combination therapies, which are not always effective. This underlines the urgent need for novel antifungal drugs, and more strict infection control measures to manage and prevent the spread of pan drug resistant *C. auris*.

A major factor in the persistence and virulence of *C. auris* is its capacity to form resistant biofilms on various surfaces, including living tissues and medical equipment (Malinovská et al. 2023). These biofilms create a shielded environment for *Candida* cells, enabling them to evade the host's immune defenses and withstand antifungal treatments. Inside the biofilm, *C. auris* cells are encased in a self-generated extracellular matrix that acts as a physical barrier, preventing drug penetration and promoting the survival of *Candida* cells even in adverse conditions. This biofilm-associated resistance makes treating *C. auris* infections significantly more difficult (Rossato and Colombo 2018).

Given the increasing global concern regarding *C. auris* as a multidrug-resistant pathogen and the inadequacy of existing treatments, there is a critical need to develop novel therapeutic strategies that can effectively combat both planktonic and biofilm-associated forms of this pathogen. New approaches, including the application of nanotechnology, are essential to overcoming the limitations of current therapies (Marena et al. 2023). ZnO nanoparticles (ZnO-NPs) possess several characteristics that make them effective against biofilms. Their small size allows them to penetrate the biofilm matrix more effectively than conventional antimicrobial agents (Ahmed and Othman 2024). Once within the biofilm, ZnO-NPs can interact directly with microbial cells, exerting antimicrobial effects through various mechanisms. One of the primary mechanisms by which ZnO-NPs disrupt biofilms is through the generation of reactive oxygen species (ROS) (Guo et al. 2013). ZnO-NPs produce ROS, such as hydroxyl radicals and hydrogen peroxide, when exposed to light or moisture. These ROS can damage microbial cell membranes, proteins, and DNA, leading to cell death and biofilm disruption. This oxidative stress can weaken the biofilm structure and enhance the susceptibility of biofilm-forming pathogens to other treatments (da Cruz Nizer et al. 2024). On the other hand, ZnO-NPs offer a promising strategy for preventing cell adhesion during biofilm formation, which is a critical initial step in biofilm development (Fayed et al. 2021). ZnO-NPs can disrupt the adhesion process through several mechanisms. For instance, ZnO-NPs can down regulate *ALS5* gene (Agglutinin-like sequence protein gene) that permit cell adhesion and biofilm formation (Fayed et al. 2021). ZnO-NPs also offer advantages in terms of biocompatibility and low toxicity (Anjum et al. 2021).

The synthesis of ZnO-NPs can be achieved through both biological and chemical methods, each with distinct advantages and considerations. Their ability to be synthesized through both biological and chemical methods allows for customization of their size, shape, and surface properties, optimizing their effectiveness for specific applications (Mandal et al. 2022; Murali et al. 2023).

Biological synthesis of ZnO-NPs is recognized for its eco-friendly and sustainable nature (El Golli et al. 2023). This method utilizes natural reducing agents, such as plant extracts, microbial systems, or enzymes, to convert zinc ions into nanoparticles (El Golli et al. 2023; Sharifabady et al. 2023). The biological approach often involves fewer hazardous chemicals and produces less toxic by-products, making it a greener alternative to traditional methods. Additionally, biological synthesis can offer a degree of natural variability and functionality, which may enhance the nanoparticles' biological interactions and applications (Huq et al. 2023; Murali et al. 2023). On the other hand, chemical synthesis provides precise control over the size, shape, and surface properties of ZnO NPs (Abdelmigid et al. 2022). This method typically involves chemical reactions using metal salts and reducing agents in a controlled environment, allowing for the production of nanoparticles with specific characteristics tailored to particular applications (Reda and Fayed 2023). Although chemical synthesis can achieve high purity and uniformity, it may involve the use of hazardous reagents and solvents, raising concerns about environmental impact and safety (Al-Mohaimeed et al. 2022).

The novelty of this study lies in the comparative evaluation of ZnO-NPs synthesized through chemical and biological methods against *C. auris*. By comparing these methods, the study aims to uncover unique advantages or limitations associated with each synthesis route, offering new insights into how these nanoparticles perform against both planktonic *C. auris* and prevent biofilm formations. This comparative analysis could reveal novel therapeutic potentials and guide the optimization of ZnO NPs use in combating resistant fungal infections.

Hence, the aim of the study is to evaluate and compare the activity of ZnO-NPs, synthesized through biological and chemical methods, against *C. auris* in its planktonic form. The study will also assess the impact of these nanoparticles on resistance genes and their effectiveness in preventing biofilm formation. Additionally, the study aims to investigate the molecular mechanisms underlying their activity. This aim is crucial because it addresses key challenges in treating *C. auris* infections. Evaluating ZnO-NPs against the planktonic form of *C. auris* will reveal their direct antimicrobial efficacy. Further, assessing their impact on resistance genes will help determine their potential to influence drug resistance. Finally, preventing *C. auris* biofilm formation is essential, as biofilms protect the pathogen and complicate treatment.

## Materials and methods

### Preparation of ZnO NPs

#### Biological synthesis of ZnO NPs

Zinc oxide nanoparticles used in this study were synthesized previously by El Sayed following the method described by (El-Sayed et al. 2021). Briefly, *Lactobacillus gasseri* were inoculated in sterile de Man, Rogosa, and Sharpe (MRS) broth and incubated at 37 °C for 24 h under mild agitation. After incubation, the pH was adjusted to 6 then, 0.1 M ZnSO₄·H₂O was added to the culture flasks serving as the precursor for ZnO-NP synthesis. The flasks were heated in a water bath at 80 °C for 10 min to facilitate the reduction of zinc ions and the nucleation of ZnO-NPs. The formation of ZnO NPs was indicated by the appearance of a white precipitate at the bottom of the flasks. The flasks were then incubated at 37 °C for an additional 12 h then the precipitated ZnO NPs were filtered, washed with distilled water, and dried at 40 °C for 4 h. The resulting ZnO NPs were characterized as described in the publication, and the same batch was utilized for the experiments in this study. The produced nanoparticles were referred to as ZnO-NPs-B.

#### Chemical synthesis of ZnO NPs

In the current study, two types of ZnO NPs were synthesized chemically. The first type, referred to as ZnO-NPs-C1, was purchased from US Research Nanomaterials, Inc. (Houston, TX, USA) and was synthesized using the dry–wet chemical method, as described in the manufacturer's manual. The second type of ZnO nanoparticles referred to as ZnO-NPs- C2, was prepared chemically by sol–gel method in stoichiometric proportions (Reda and Fayed 2023). The gel formation involved the use of citric acid (C_6_H_8_O_7_⋅H_2_O) and polyethylene glycol in a 60:40 mass% ratio as a crosslinking agent. The process began by dissolving citric acid in deionized water for 15 min. Next, stoichiometric quantities of Zn(CH_3_COO)_2_·2H_2_O, was dissolved in deionized water while stirring continuously. Once the salts were completely dissolved in the citric acid solution, polyethylene glycol was added. The mixture was then heated at 110 °C for 1 h with constant stirring to evaporate most of the water and initiate gel formation. The resulting gel was dried at 60 °C for 24 h, producing the ZnO-NPs- C2 nanoparticle. These dried gels were calcined at 600 °C for 1 h in air. X-ray diffraction (XRD) analysis was employed to investigate the phases present in the calcined ZnO-NPs- C2 sample heated at 600 °C for 1 h. The analysis was performed using a D8 Advance diffractometer (Bruker, Germany) equipped with a secondary monochromatic CuKα radiation beam, operating at 40 kV and 40 mA. The crystalline phase of the nanoparticle formed at 600 °C were identified, with intensity data recorded over a 2θ range from 10° to 60°. Further, the surface morphology of the calcined ZnO-NPs- C2 sample was examined using SEM. After being sputter-coated with gold, the sample was analyzed with a Philips XL30 scanning electron microscope. The SEM operated at an accelerating voltage of 30 kV and provided magnifications of up to 400,000x, capturing detailed photomicrographs of the sample’ surface structure.

### Assessment of particle size and charge

The particle size and polydispersity index (PDI) of ZnO-NPs were determined at ambient temperature using photon correlation spectroscopy (PCS) with a detection angle of 90°, employing a 90 Plus Particle Size Analyzer (Brookhaven Instruments Corporation, Holtsville, NY, USA). The PDI was used to assess the uniformity of the particle size distribution, with lower values indicating more uniform particle sizes. In addition, the surface charge (zeta potential) of the ZnO NPs were measured using photon correlation spectroscopy with a 90 Plus Zetasizer (Brookhaven Instruments Corporation, Holtsville, NY, USA). Zeta potential measurements were conducted to evaluate the colloidal stability of the nanoparticles in suspension. All measurements were performed in triplicate to ensure data reliability, and the results were reported as mean ± standard deviation.

### Antifungal susceptibility testing

Antifungal susceptibility testing of ZnO-NPs was carried out against a *C. auris* strain, clinical isolate obtained from the Centers for Disease Control and Prevention (CDC), Atlanta, Georgia, USA**,** following the method described by (Ismail et al. 2024). An overnight culture of *C. auris* in Sabouraud Dextrose broth media (SD) was adjusted to 10^4^ CFU/ml, and serial dilutions of ZnO-NPs were added (400, 200, 100, 50, 25, 12.5 µg/ml). The cultures were incubated at 37 °C for 48 h in flat-bottom 96-well plates (Costar, USA). After incubation, *C. auris* growth was assessed using a microplate reader at OD_600_. The data were used to calculate the MIC_50_, defined as the minimum nanoparticle concentration that resulted in a 50% reduction in fungal growth compared to the untreated control. All tests were performed in triplicate, and the average MIC_50_ value was recorded. Free media, media contains untreated *C. auris,* and media contains *C. auris* treated with 100 ng/ml caspofungin were used as controls.

### Gene expression analysis of resistance genes

Cells of *C. auris* at 10^6^ CFU/ml were incubated with each of the three ZnO-NPs formulations at their respective MIC_50,_ except ZnO-NP-B that was used at 1 mg/ml, for 48 h in SD media (Fayed et al. 2021). Following incubation, the cells were obtained by centrifugation at 4000 rpm for 10 min at 4 °C, washed then disrupted using glass beads for 10 min. Following centrifugation at 4 °C for 20 min, RNA was extracted from the resulting supernatant using a total RNA purification kit (NORGEN, Auburn, WA) following the manufacturer’s guidelines. RNA concentration and quality were evaluated using a NanoDrop 2000 spectrophotometer (Thermo Fisher Scientific, USA). cDNA synthesis was carried out from 1 μg of RNA using the RevertAid First Strand cDNA Synthesis Kit (Thermo Fisher Scientific, Lithuania).

Quantitative real-time PCR was performed to detect the level of expression of resistance genes (*CDR1, MDR1, FKS1, ERG2, ERG11, CHS1*) with the Hera Plus SYBR Green qPCR Kit (Willow Fort, Birmingham, UK) according to the manufacturer’s instructions to assess mRNA expression levels of target genes. The PCR plates were analyzed with the ABI 7500 Fast System (Applied Biosystems), and melting curve analysis was used to verify the specificity of the amplification. Gene expression fold changes were calculated using the comparative threshold cycle method (2^−ΔΔCt^), with normalization to the endogenous control (*ACT1* gene) (Livak and Schmittgen 2001). Untreated cells were used as control. Primer sequences used in the study are listed in Table 1.

**Table 1:** List of primers used in the study

Gene name	Gene symbol	Primer sequence (5′–3′)
Actin gene	*ACT1*	FW-CGCTGGTTTCTCGTTACCAC RV-CAGCAGTGGTAGAGAAGGTGT
Lanosterol 14-alpha-demethylase synthase gene	*ERG11*	FW-CAAGTCGTTGATGGGTGATG RV-GAACGATGTCACCGGTCTTT
Gene encode C-8 sterol isomerase	*ERG2*	FW-GAGAGGCCAAGTGAAGCAGT RV-ACACAAAGCCGAATGGCAAC
1,3-beta-D-glucan synthase gene	*FKS1*	FW-CGAAGAACACGGTCAGGACA RV-CCTCAGGGGTCAAGACGTTC
Chitin synthase I gene	*CHS1*	FW-CGCCGTTTACAACCTTTGGA RV-TGAGAAGCAACGAGTGGGTTT
Gene encodes ABC multidrug transporter MDR1	*MDR1*	FW-TCAGACACCCCCGTTGATCT RV-TCTTCTCCCTCCCAGTCCAC
Gene encodes efflux pump gene *CDR1*	*CDR1*	FW-GCCAGGTTTCTGGATTTTCA RV-GGCCACAAGTTTGACCACTT
Agglutinin-like sequence protein gene *ALS5*	*ALS5*	FW-CCTTCTGGATCGGACACAGT RV-AGTTGTGGTGGAGGAACCAG
Gene encodes GPI-anchor protein *IFF4*	*IFF4*	FW-CTCAACATCAACCCTGGCGT RV-AGAAGCAGCCAATCCGTTGT
Gene encodes a mycelial surface antigen	*CSA1*	FW-TCCTCCAAGAACATCCGCAC RV-GAGGAAGTGGCTCTGGTGAC

### Evaluation of ZnO-NPs in preventing *C. auris* biofilm formation at the adhesion stage

The three formulations of ZnO-NPs were incubated with *C. auris* at 150 µg/ml. The fungal cells were adjusted to 10^6^ CFU/ml in SD media. The incubation was performed in a 96-well plate for 4 h at 37 °C. After incubation, non-adherent cells were removed, and the adhered cells were washed twice with sterile PBS to remove the NPs then fresh media were added and the adhered cells were further incubated for 24 h. Biofilm formation following the incubation period was assessed using the XTT assay as follows. The adhered cells were washed twice with PBS, followed by the addition of 1 ml of 5 mg/mL XTT (2,3-(2-methoxy-4-nitro-5-sulphophenyl)-5-[(phenylamino)carbonyl]-2H-tetrazolium hydroxide) and 1 mM menadione (Osman et al. 2023). The plate was incubated in the dark at 37 °C with shaking at 100 rpm. Once a color change occurred, 100 μl of the solution was transferred to a 96-well plate, and absorbance was measured at 490 nm using a microplate reader. All assays were performed in triplicate, and the average absorbance was recorded. Untreated cells, free media and media contains 100 ng/ml caspofungin were used as controls.

### Gene expression analysis for *C. auris* adhesive genes

*C. auris* cells, adjusted to 10^6^ CFU/mL, were incubated in SD medium with ZnO-NP-C1 at 150 µg/ml, in a 6-well plate at 37 °C for 4 h. Following the incubation period, non-adherent cells were collected, and washed twice with sterile PBS. RNA extraction and quantitative real-time PCR was performed as mentioned earlier for adhesive genes (*ALS5*, *IFF4, CSA1*) using primer listed in Table 1. Untreated cells were used as negative control and the experiment was performed in replicates.

### Statistical analysis

The MIC_50_ was determined and plotted using GraphPad Prism (version 8.02, GraphPad Inc., La Jolla, CA, USA). For the gene expression analysis, an unpaired t-test with Welch correction was applied. A p-value of less than 0.05 was considered statistically significant. Results are shown as mean ± SD from three independent replicates.

## Results

### Preparation and characterization of ZnO-NP-C2

**Fig. 1:**
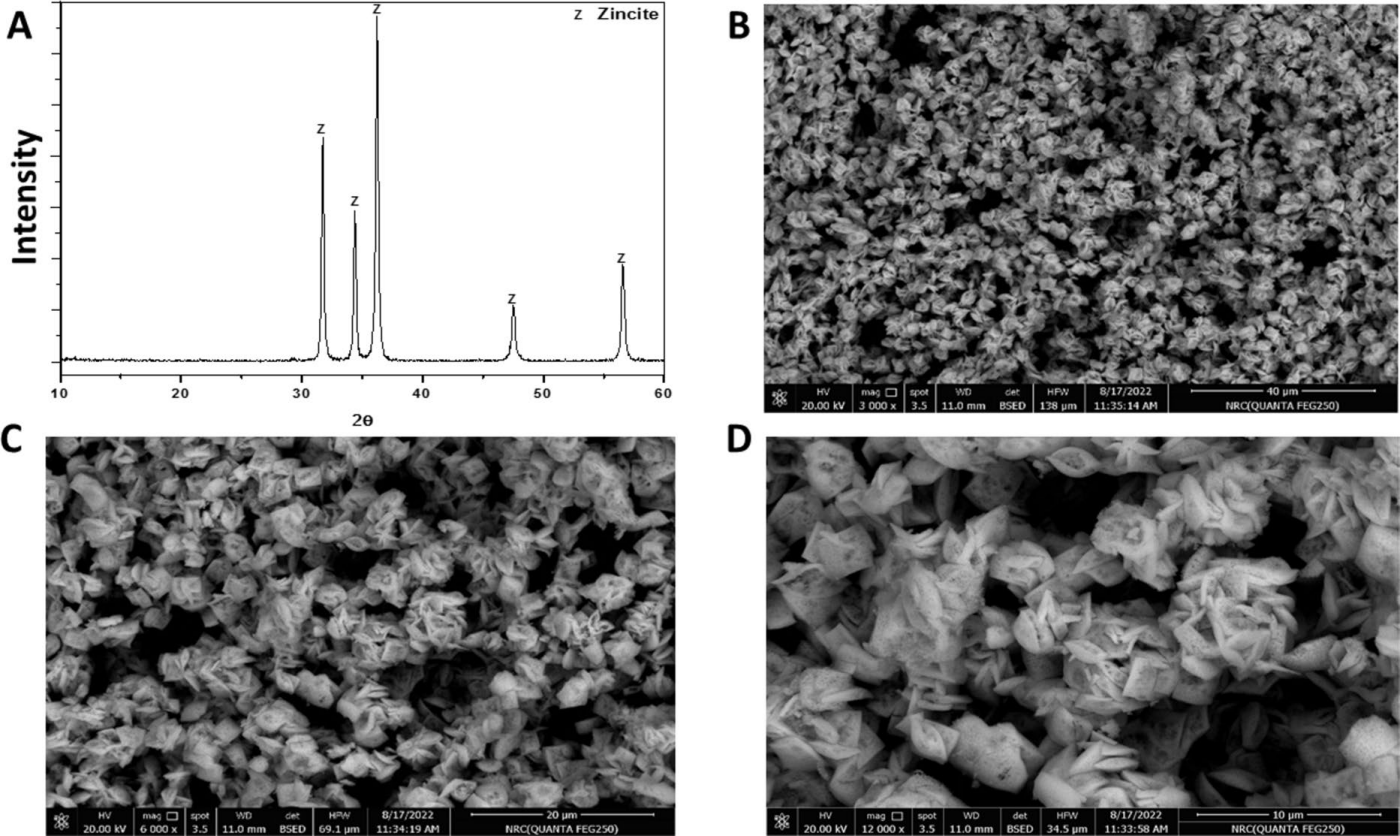
Characterization of chemically synthesized ZnO-NP-C2 Nanoparticles. **A** XRD pattern showing the hexagonal wurtzite crystalline structure of the synthesized ZnO-NP-C2. **B**–**D** SEM images depicting nanoflake-like morphology and plate-like structures formed by ZnO-NP-C2

The ZnO-NP-C2 were synthesized, and their structural properties were characterized through XRD and SEM. The XRD pattern of ZnO-NP-C2 exhibited intense and well-defined peaks, indicative of the hexagonal wurtzite crystalline structure, confirming ZnO as the dominant phase with high crystallinity (Fig. 1A). These peaks correspond to the standard ICDD card (01–074–3156), confirming the formation of pure ZnO nanoparticles without detectable impurities. The SEM analysis revealed that ZnO-NP-C2 particles adopted a distinct nanoflake-like morphology, with individual flakes forming plate-like structures as indicated from Fig. 1B–D.

### Comparison of particle size and zeta potential of chemically and biologically synthesized ZnO-NPs

**Fig. 2:**
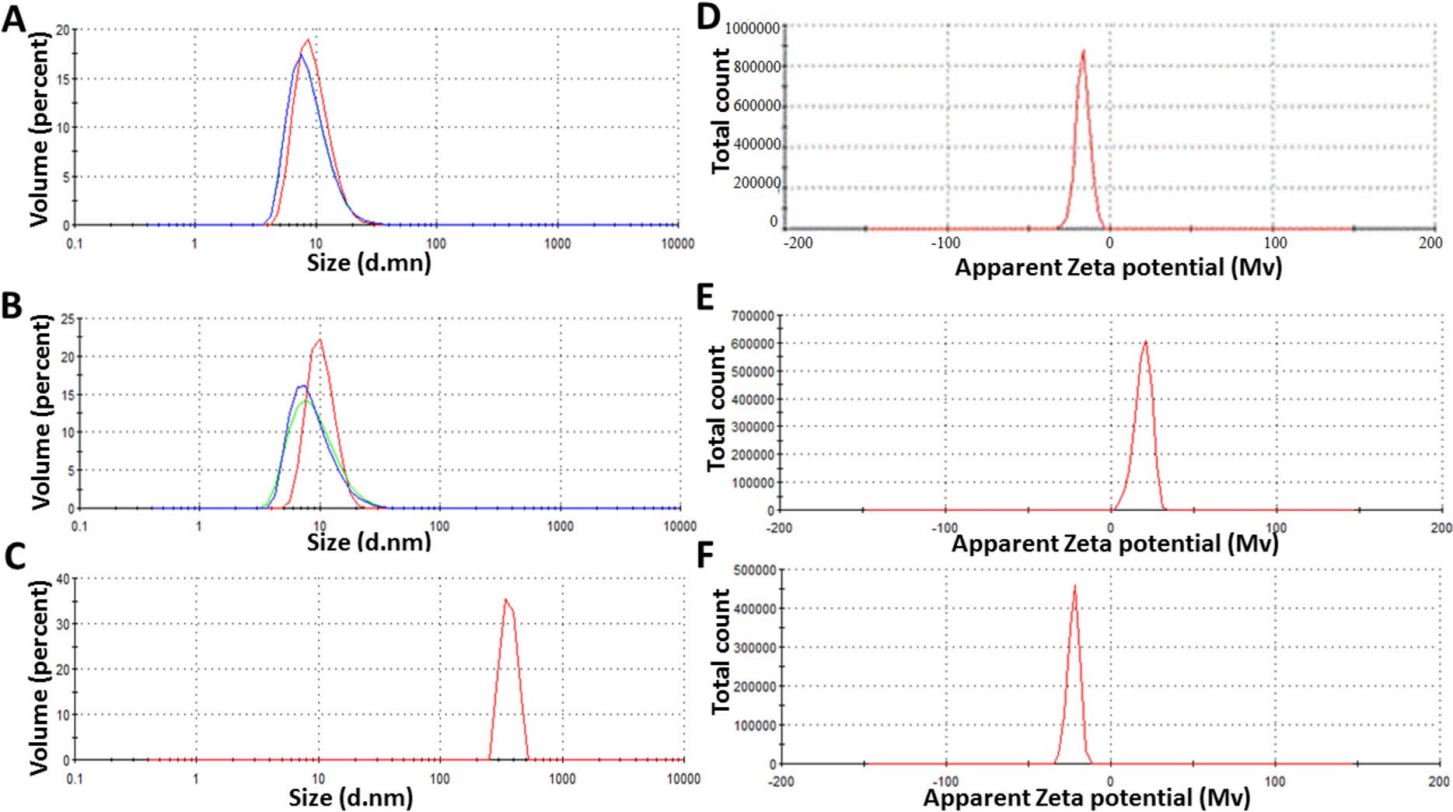
Comparison of particle size and surface charge of biologically and chemically synthesized ZnO Nanoparticles. **A** ZnO-NP-C1 showing an average size of 9.62 ± 3.2 nm. **B** ZnO-NP-C2 with an average size of 10.36 ± 2.65 nm. **C** ZnO-NP-B synthesized using biological methods with a larger particle size of 604.2 ± 374.6 nm. **D**–**F** Zeta potential measurements indicating differences in surface charge: ZnO-NP-C1 (− 25.8 mV), ZnO-NP-C2 (+ 19.4 mV), and ZnO-NP-B (− 22.6 mV)

The ZnO-NPs synthesized using both biological and chemical methods were extensively characterized in terms of their particle size and zeta potential. The data obtained, as illustrated in Fig. 2, demonstrated significant variations between the different synthesis methods, indicating that the synthesis approach has a marked influence on the physicochemical properties of the resulting NPs. The smallest particle size was observed in the chemically synthesized ZnO-NP-C1, and ZnO-NP-C2 with an average size of 9.62 ± 3.2 nm and 10.36 ± 2.65 nm, respectively (Fig. 2A, B). In contrast, ZnO-NP-B, synthesized using a biological approach, exhibited the largest particle size, with an average of 604.2 ± 374.6 nm (Fig. 2C). The zeta potential measurements provided further insight into the surface charge and colloidal stability of the nanoparticles in suspension. ZnO-NP-C1 exhibited a zeta potential of -25.8 mV, ZnO-NP-C2, in contrast, had a zeta potential of + 19.4 mV, indicating a positive surface charge (Fig. 2D, E). Further, ZnO-NP-B, synthesized biologically, had a zeta potential of -22.6 mV (Fig. 2F).

### Chemically synthesized ZnO nanoparticles exhibit potent activity against *C. auris* compared to biologically synthesized nanoparticles

**Fig. 3:**
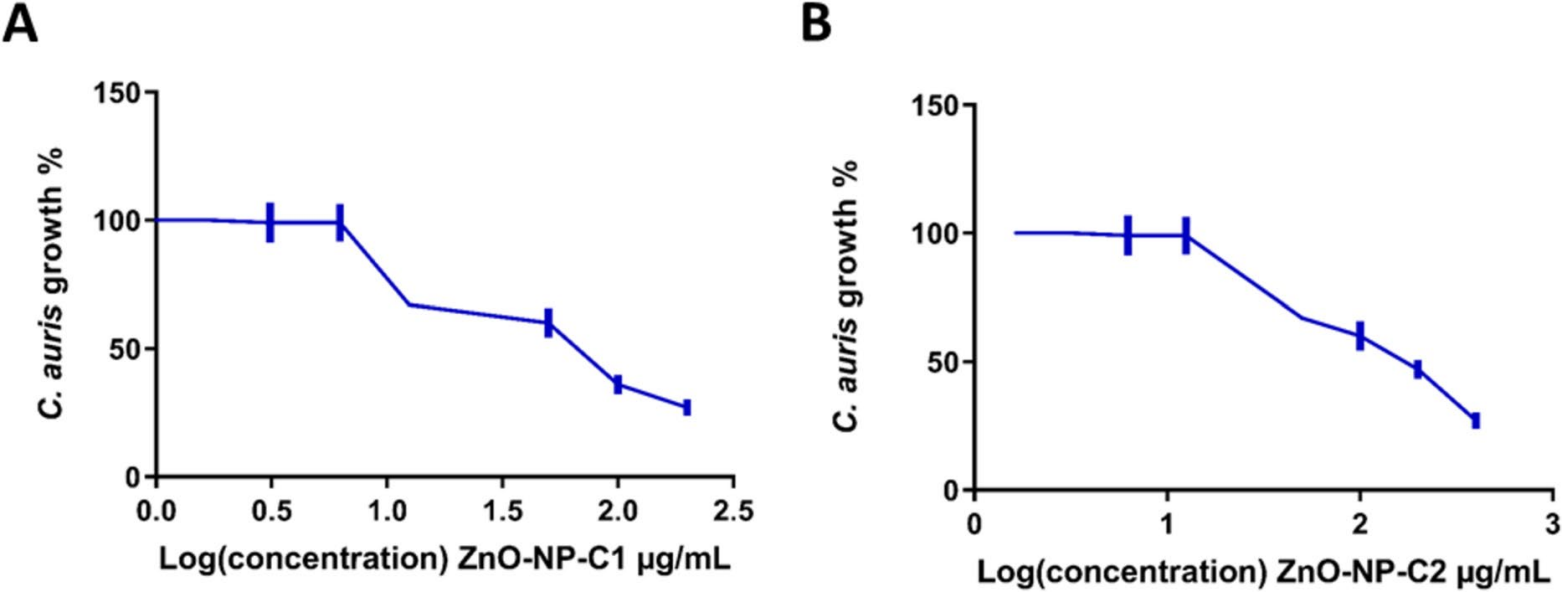
Antifungal activity of chemically synthesized ZnO Nanoparticles against *C. auris*. **A** Activity of ZnO-NP-C1. **B** Activity of ZnO-NP-C2

Antifungal susceptibility testing revealed a significant difference in the activity of chemically and biologically synthesized ZnO-NPs against *C. auris*. Both chemically synthesized ZnO-NP-C1 and ZnO-NP-C2 demonstrated strong antifungal effects on *C. auris* planktonic cells, with MIC_50_ of 61.9 ± 3.3 µg/ml and 151 ± 7.83 µg/ml, respectively (Fig. 3A, B). In contrast, biologically synthesized ZnO-NP-B exhibited weak antifungal activity at concentrations 1 mg/ml.

### Differential gene expression response of *C. auris* to chemically and biologically synthesized ZnO nanoparticles

**Fig. 4:**
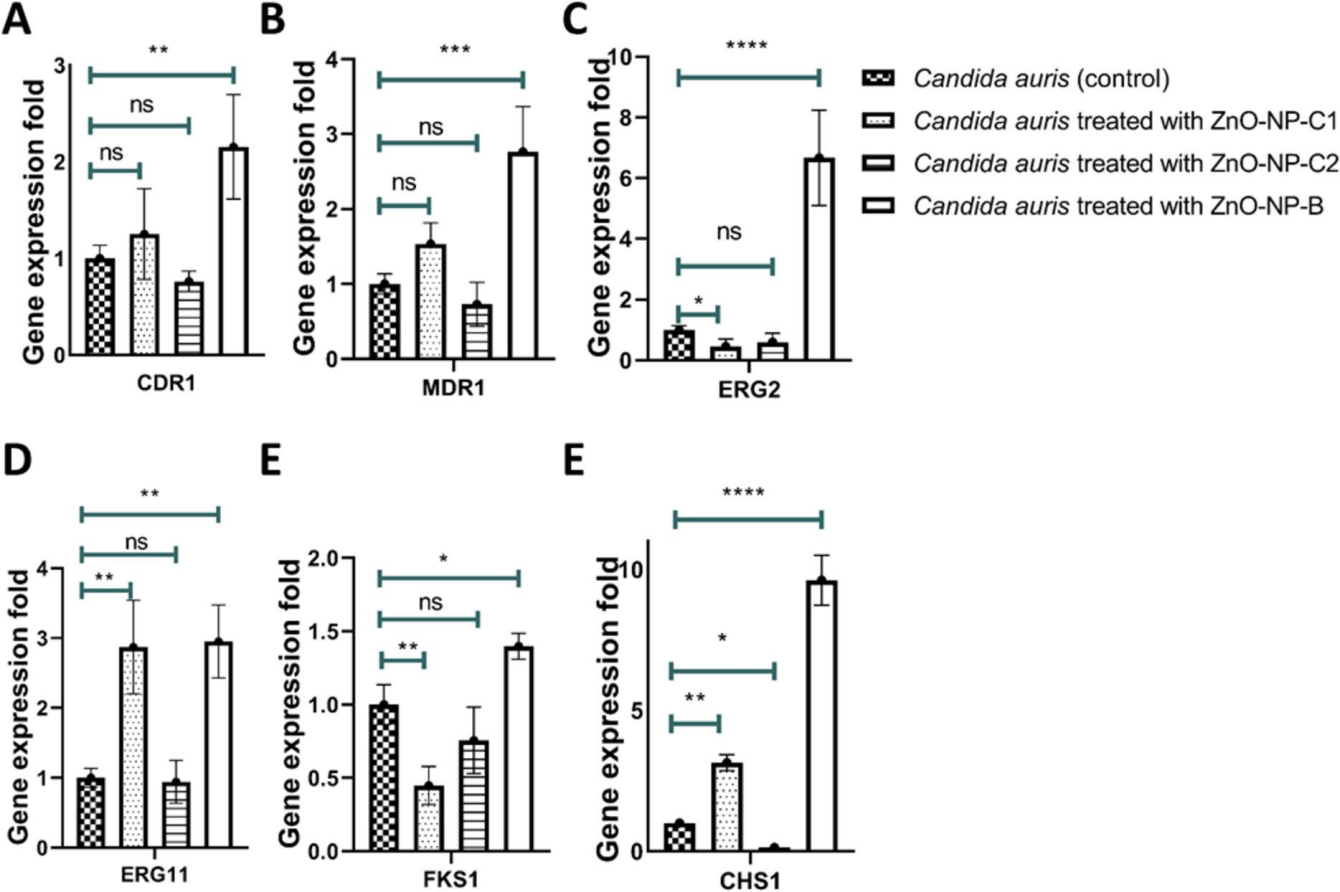
Effect of ZnO Nanoparticles on the expression of antifungal resistance genes in *C. auris*. **A** Gene expression analysis of *CDR1* gene, **B** Gene expression analysis of *MDR1* gene, **C** Gene expression analysis of *ERG2* gene, **D** Gene expression analysis of *ERG11* gene, **E** Gene expression analysis of *FKS1* gene, **F** Gene expression analysis of *CHS1* gene

Our data, as shown in Fig. 4 demonstrate a striking difference in the impact of biologically synthesized ZnO-NP-B and chemically synthesized ZnO-NP-C1 and ZnO-NP-C2 on the expression of antifungal resistance genes in *C. auris*. ZnO-NP-B significantly upregulated the expression of several key resistance genes. The fold changes in expression were as follows: *CDR1* (2.15 ± 0.54), *MDR1* (2.76 ± 0.6), *ERG2* (6.6 ± 1.57), *ERG11* (2.95 ± 0.52), *FKS1* (1.39 ± 0.088), and *CHS1* (9.6 ± 2.88). On the other hand, treatment with chemically synthesized ZnO-NP-C2 did not affect the expression of the same set of resistance genes with the exception of *CHS1* that downregulated with fold change (0.13 ± 0.03). Interestingly, ZnO-NP-C1 exhibited a mixed effect on gene expression. While *ERG11*, and *CHS1* were overexpressed, with fold changes of 2.87 ± 0.67, and 3.14 ± 0.28 respectively, *ERG2* and *FKS1* were downregulated to 0.45 ± 0.24 and 0.44 ± 0.12, respectively, with no significant effect on *CDR1* and *MDR1* expression.

### Effect of ZnO Nanoparticles on preventing *C. auris* adhesion and early biofilm formation

**Fig. 5:**
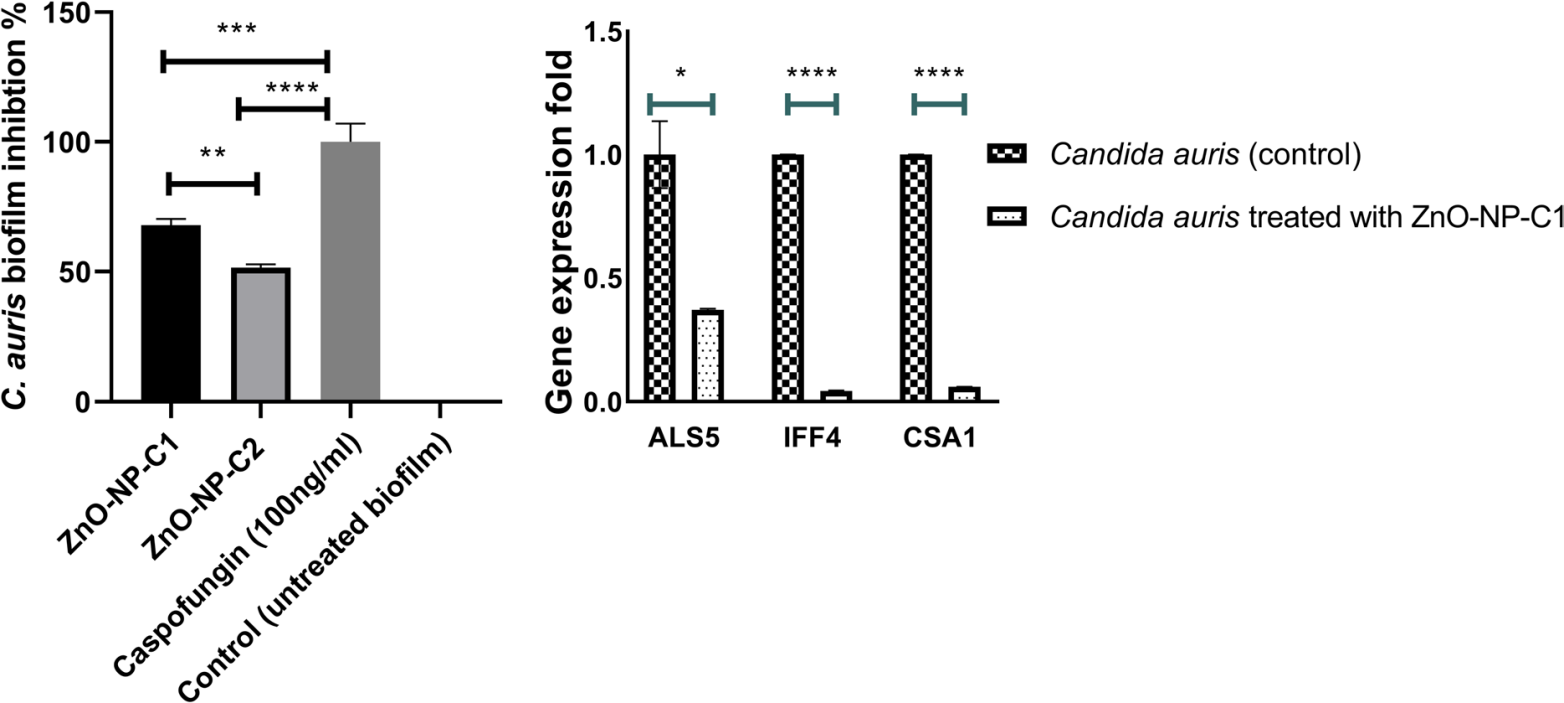
Inhibition of *C. auris* adhesion and biofilm formation by chemically synthesized ZnO Nanoparticles. **A** XTT analysis for *C. auris* biofilm treated with ZnO-NP-C1, ZnO-NP-C2, and caspofungin 100 ng/ml. **B** Gene expression analysis for *C. auirs* adhesive genes following treatment with ZnO-NP-C1

We evaluated the ability of ZnO-NPs to inhibit the adhesion of *C. auris* by incubating the fungal cells with the nanoparticles for 4 h. As shown in Fig. 5A, ZnO-NP-C1 demonstrated 67.9 ± 2.35% inhibition of *C. auris* adhesion, effectively preventing the fungal cells from attaching to the surface. ZnO-NP-C2 also exhibited significant antifungal activity, showing a strong inhibitory effect on *C. auris* adhesion, though not to the same extent as ZnO-NP-C1 as the inhibition was 51.6 ± 1.26%. In contrast, biologically synthesized ZnO-NP-B1 had no measurable effect on the adhesion of *C. auris*.

### Molecular mechanism of ZnO-NP-C1 in inhibiting *C. auris* adhesion

To investigate the molecular mechanism by which ZnO-NP-C1 inhibits *C. auris* adhesion and early biofilm formation, gene expression analysis was performed on three key protein-encoding genes: *ALS5*, *IFF4,* and *CSA1*. As shown in Fig. 5B, the data revealed a substantial downregulation of all the targeted adhesive genes compared to the untreated control. The expression levels of *ALS5*, *IFF4 and CSA1*, which are critical for initial adhesion, were notably reduced, with values of 0.37 ± 0.006, 0.043 ± 0.002 and 0.06 ± 0.0004, respectively.

## Discussion

This study offers a thorough comparison of biologically and chemically synthesized ZnO-NPs in terms of their antifungal activity against *C. auris* and their ability to prevent biofilm formation. The findings demonstrate that ZnO-NPs, particularly those synthesized chemically (ZnO-NP-C1 and ZnO-NP-C2), exhibit potent antifungal activity and the capacity to inhibit biofilm formation, thus offering a promising alternative to conventional antifungal treatments.

The characterization of ZnO-NPs synthesized through biological and chemical methods reveals significant differences in their physicochemical properties, which likely influence their antimicrobial activity. For example, chemically synthesized ZnO-NP-C1 and ZnO-NP-C2 exhibit much smaller particle sizes, which is advantageous for antimicrobial activity due to the increased surface area-to-volume ratio. This enhancement facilitates better interaction and penetration into microbial cells (Mondal et al. 2024). In contrast, biologically synthesized ZnO-NP-B has a much larger average particle size, which may hinder its ability to diffuse through cell membranes and interact effectively with microbial cells, potentially reducing its antimicrobial efficacy. This is supported by findings from Dong et al., who demonstrated that smaller silver nanoparticles (Ag-NPs) exhibited lower MICs and greater antibacterial activity compared to larger particles, highlighting the critical role of NPs size in antimicrobial effectiveness. Further research has also emphasized the importance of NPs size on antimicrobial activity, particularly for ZnO NPs. Smaller ZnO nanoparticles were found to exhibit superior antimicrobial efficacy compared to their larger counterparts (Dong et al. 2009). The correlation between nanoparticles size/morphology and antifungal efficacy extends beyond planktonic activity to biofilm inhibition (Fakeeha et al. 2024). The superior performance of ZnO-NP-C1 in preventing *C. auris* adhesion can be attributed to its small particle size, which enhance interaction with fungal cell surfaces. ZnO-NP-C2, despite its nanoflake morphology, also exhibited strong inhibition on *C. auris* adhesion and consequently biofilm formation. Conversely, the biologically synthesized ZnO-NP-B failed to prevent *C. auris* adhesion, likely due to its larger size reducing its ability to penetrate cell surface and effectively interfere with fungal adhesion mechanisms. These findings underscore the importance of nanoparticle size in optimizing antifungal efficacy and biofilm inhibition (Fakeeha et al. 2024).

In addition to particle size, zeta potential measurements provide insights into the NPs surface properties and colloidal stability. ZnO-NP-C1 and ZnO-NP-B both have a negative zeta potential with higher magnitude, indicating good colloidal stability (Kuyukina et al. 2022). On the other hand, ZnO-NP-C2 has a high positive zeta potential, suggesting a positive surface charge that could enhance adhesion to negatively charged microbial membranes and disrupt cell wall integrity (Kuyukina et al. 2022; Arakha et al. 2015). Hence, these surface charge differences together with the obtained particle size contribute to the observed variations in antimicrobial activity among the different ZnO-NPs prepared in this study. Also, it can be indicated that the chemical synthesis method offers a significant advantage in controlling nanoparticle size compared to biological methods that provides a substantial advantage in enhancing antimicrobial activity (Vishwanath and Negi 2021). Moreover, considering the antimicrobial properties of ZnO-NPs, several reports highlighted that the exposure to antifungal agents often leads to the upregulation of resistance genes and adaptive responses that enable *C. auris* to survive despite treatment (Fayed et al. 2021; Alatoom et al. 2018). Hence it was crucial to explore the effect of exposing *C. auris* to ZnO-NPs on the expression of *Candida* resistance genes. The resistance genes selected for this study include *CDR1, MDR1, ERG2, ERG11, CHS1* and, *FKS*. Genes involved in membrane transport, such as the ABC transporter gene *CDR1* and the major facilitator gene *MDR1*, are central to resistance development in *Candida* species (Prasad et al. 2015; Wirsching et al. 2000). Overexpression of these genes has been associated with resistance to fluconazole and caspofungin (Fayed et al. 2021). For instance, *C. albicans* strains resistant to fluconazole showed elevated expression of *CDR1, CDR2*, and *MDR1* (Khosravi Rad et al. 2016). Comparable findings were reported by EL-Kholy et al. (El-Kholy et al. 2023). Additionally, *C. auris* developed multidrug resistance by upregulating *CDR1* expression following exposure to antifungal drug (Fayed et al. 2021). Previous research by Ahmad et al. identified that *ERG2* is contributing to reduced susceptibility to amphotericin B in *Candida glabrata* (Ahmad et al. 2019). Alterations or increased expression of *ERG2* interfere with ergosterol synthesis, the primary target of amphotericin B, thus reducing its effectiveness. The *ERG11* gene encodes lanosterol demethylase, the enzyme targeted by azole antifungals, so mutations or overexpression of *ERG11* reduce the effectiveness of azoles (Fayed et al. 2021). For example, overexpression of *ERG11* has been shown to increase the MIC_50_ for fluconazole by threefold in *C. auris* (Fayed et al. 2021), similar observation was noted in *C. albicans* (Morais Vasconcelos Oliveira et al. 2021). Further, overexpression of *CHS1* and *FKS1* has been identified as a key mechanism driving resistance to echinocandins in *Candida* species [28, 29]. The *CHS1* gene encodes chitin synthase, while *FKS1* encodes 1,3-β-d-glucan synthase [29, 30]. In our previous research, overexpression of these genes increased caspofungin resistance [2]. Herein, the data obtained highlight significant differences in the impact of biologically synthesized ZnO-NP-B1 and chemically synthesized ZnO-NP-C1 and ZnO-NP-C2 on the expression of antifungal resistance genes in *C. auris*. Biologically synthesized ZnO-NP-B1 notably upregulated the expression of several key resistance genes. This suggests a robust induction of resistance mechanisms. In contrast, chemically synthesized ZnO-NP-C2 induced a downregulation of the same set of resistance genes. This suggests that ZnO-NP-C2 might lower the resistance mechanisms of *C. auris*, potentially making it more susceptible to antifungal treatments. ZnO-NP-C1 displayed a mixed effect on gene expression that implies ZnO-NP-C1’s impact on resistance genes may vary depending on the specific gene or pathway.

ZnO-NPs modulate *C. auris* resistance genes through oxidative stress, membrane disruption, and cell wall interference. In response to these stresses, *C. auris* activates protective mechanisms by upregulating genes involved in efflux pump activity, ergosterol biosynthesis, and cell wall integrity (Fayed et al. 2021). The degree of this upregulation determines the extent of resistance, as higher expression of these genes enhances the fungus’s ability to counteract ZnO-NP-induced damage. The generation of ROS triggers the upregulation of efflux pump genes (*CDR1*, *MDR1*), enhancing drug efflux and resistance (Schubert et al. 2011). Membrane disruption will trigger the expression of *ERG2* and *ERG11*, affecting membrane integrity (Fayed et al. 2021). Additionally, membrane disruption can trigger the production of β-glucan and chitin by overexpressing *FKS1* and *CHS1* genes (Fayed et al. 2021). Interestingly, we observed in a previous study a similar pattern when *C. auris* was exposed to caspofungin loaded onto ZnO-NPs, as the fungus failed to overexpress resistance genes to develop resistance to caspofungin in its nanoparticle-loaded form compared to exposure to free caspofungin (Fayed et al. 2021). This suggests that ZnO-NPs can interfere with adaptive resistance mechanisms, potentially enhancing the efficacy of antifungal treatments.

The gene expression analysis of key adhesive genes further supports the efficacy of ZnO-NP-C1 in inhibiting *C. auris* adhesion and early biofilm formation. The substantial downregulation of *ALS5*, *IFF4*, and *CSA1*, which are critical for initial adhesion, demonstrates that ZnO-NP-C1 effectively disrupts the very first steps in biofilm establishment (Kean et al. 2018). This molecular data correlates well with the observed inhibition of *C. auris* adhesion in our earlier experiments, where ZnO-NP-C1 proved to be the most potent in blocking cell attachment compared to other formulations. We selected ZnO-NP-C1 for these molecular studies specifically because of its superior performance in preventing *C. auris* adhesion. These findings further highlight the broader implications of ZnO-NP physicochemical properties on fungal resistance mechanisms. The downregulation of *ALS5*, *IFF4*, and *CSA1* also suggests a broader antifungal effect beyond adhesion inhibition. These genes encode cell wall proteins that are essential for fungal adherence, biofilm maturation, and persistence on surfaces (Kempf et al. 2009). Previous transcriptomic studies have shown that these adhesin-related genes are upregulated at all stages of *C. auris* biofilm development, reinforcing their role in biofilm integrity and antifungal resistance (Kean et al. 2018). By suppressing their expression, ZnO-NP-C1 likely prevents fungal attachment, thereby disrupting biofilm initiation and reducing the overall pathogenic potential of *C. auris*. Moreover, these genes function as virulence factors, contributing to fungal colonization, immune evasion, and persistence on host tissues and medical devices. *ALS5* is homologous to ALS family adhesins in Candida albicans, which facilitate host cell adherence, while *IFF4* and *CSA1* play crucial roles in biofilm integrity and antifungal resistance (Kean et al. 2018; Fu et al. 2008). Their downregulation suggests a promising strategy for developing antifungal coatings on medical devices, as preventing fungal adhesion at the molecular level could significantly reduce biofilm-associated infections.

Further, several studies have reported the synthesis of ZnO-NPs using green chemistry approaches, demonstrating both antibacterial and antifungal activities (Khan et al. 2021; Kang et al. 2022; Fattahi et al. 2025). For instance, ZnO-NPs synthesized using phloroglucinol have shown antibiofilm and antivirulence activities against *Pseudomonas aeruginosa* PAO1(Khan et al. 2021). Our study differs from these reports in several key aspects. While previous studies have focused on bacterial pathogens like *P. aeruginosa*, our research specifically targets *C. auris*, a multidrug-resistant fungal pathogen known for its ability to form robust biofilms and cause invasive infections. Additionally, our study explore the molecular mechanisms underlying the antifungal activity of ZnO-NPs against *C. auris*, including their impact on gene expression related to adhesion, biofilm formation, and resistance. This provides a deeper understanding of how ZnO-NPs interact with fungal cells at the molecular level. Another key distinction is our finding that ZnO-NP-B can induce adaptive resistance mechanisms in *C. auris*, highlighting the need to optimize nanoparticle design to prevent unintended resistance development, a factor that may not have been thoroughly addressed in previous studies.

These findings underscore the necessity for tailored nanoparticle designs when targeting specific pathogens, especially those with complex resistance profiles like *C. auris*.

While our in vitro findings demonstrate the strong antifungal and anti-biofilm activity of ZnO-NP-C1 against *C. auris*, additional studies are necessary to evaluate its translational potential. Future research should include in vivo models, such as murine or rabbit models of *C. auris* infections, to assess its efficacy, and safety under physiological conditions. Testing ZnO-NP-C1 against multiple clinical *C. auris* isolates from different clades with varying resistance profiles is also crucial to determine its broad-spectrum effectiveness, as the genetic and phenotypic diversity of *C. auris* may influence nanoparticle activity. The use of a single *C. auris* strain in this study presents a limitation, as it may not fully represent the variability in antifungal resistance, biofilm formation, and virulence seen among clinical isolates. Expanding future studies to include a wider range of *C. auris* strains will improve the generalizability of ZnO-NP efficacy and provide deeper insights into strain-specific responses. Additionally, cytotoxicity and biocompatibility studies on human cells will be essential to ensure its safety for medical applications. Investigating its impact on host immune responses and optimizing its formulation for medical device coatings or antifungal therapies will further support its potential clinical use. Another important consideration is the ability of ZnO-NPs to disrupt bacterial-fungal multi-species biofilms, as *C. auris* is frequently co-isolated with bacterial pathogens like *Staphylococcus aureus* and *Pseudomonas aeruginosa* in healthcare settings. These mixed biofilms are more resistant to antimicrobials and contribute to persistent infections. Given the broad-spectrum antimicrobial activity of ZnO-NPs, they may also be effective against polymicrobial biofilms, but this hypothesis needs validation through co-culture biofilm models. Addressing these gaps will provide a more comprehensive understanding of the clinical potential of ZnO-NPs in combating *C. auris* and complex biofilm-associated infections.

## Conclusion

This study demonstrates the superior antifungal efficacy of chemically synthesized ZnO nanoparticles (ZnO-NP-C1 and ZnO-NP-C2) over biologically synthesized ZnO-NP-B against *C. auris* biofilms. The chemically synthesized nanoparticles not only displayed potent antimicrobial activity against *C. auris* but also effectively downregulated key resistance and adhesion genes, indicating a significant potential to disrupt biofilm formation and enhance susceptibility to antifungal agents. In contrast, biologically synthesized ZnO-NP-B exhibited limited antifungal efficacy and upregulated resistance genes, suggesting potential induction of adaptive resistance. The physicochemical properties of the nanoparticles, influenced by the synthesis method, played a crucial role in determining their antifungal effectiveness. Future research should focus on in vivo studies to validate these findings and explore the clinical applicability of chemically synthesized ZnO-NPs as antifungal coatings for medical devices. This study highlights the promise of nanotechnology-based strategies in combating multidrug-resistant pathogens and addressing the challenges of biofilm-associated infections.

## Data Availability

Not Applicable.
